# Low Lactic Acid-Producing Strain of *Lachancea thermotolerans* as a New Starter for Beer Production

**DOI:** 10.3390/biom10020256

**Published:** 2020-02-07

**Authors:** Marek Zdaniewicz, Paweł Satora, Aneta Pater, Sylwia Bogacz

**Affiliations:** Department of Fermentation Technology and Microbiology, Faculty of Food Technology, University of Agriculture in Krakow, 31-120 Krakow, Poland; pawel.satora@urk.edu.pl (P.S.); a.pater@urk.edu.pl (A.P.); sylwiabogacz123@wp.pl (S.B.)

**Keywords:** beer, wort, fermentation, FAN, yeast, *non-Saccharomyces*, *Lachancea thermotolerans*

## Abstract

Growing consumer interest in new beer flavors is contributing to the application of innovative materials and non-*Saccharomyces* yeast in brewing. The goal of this study was to test the impact of the low lactic acid-producing *Lachancea thermotolerans* MN477031 strain on the process of fermenting beer wort, with two different concentrations of bitter compounds, and on the quality of the beer produced. Qualify factors were broadly analyzed, including ethanol content, apparent degree of fermentation, sugars, organic acids, free amino nitrogen, glycerol, volatile compounds, ions and so on. It was proven that the *L. thermotolerans* MN477031 strain demonstrated a high capacity for rapid initiation of wort fermentation, and a tolerance to hop-derived compounds. As a result, the alcohol content in beer from this method of production was approximately 20% lower, while the content of the real extract was significantly higher in comparison to commercial Safbrew T-58. This strain stands out from many strains of *L. thermotolerans* due to the low lactic acid production and only marginal influence on pH decrease compared to *Saccharomyces cerevisiae.* Therefore, the potential of MN477031 in the production of different types of beer (not only sour) is very high. The composition of volatile compounds in *L. thermotolerans* beer differs—not only in terms of the use of the strain, but also in hop variety.

## 1. Introduction

A growing interest in craft beer and its production has contributed to an increase in the number of microbreweries. This trend is particularly very strong in Europe [1] and the USA [2]. According to the Alltech Survey [3], in 2017, out of over 19,000 breweries around the world, craft breweries represented 94%. They owe their popularity largely to their wide variety and original styles on offer. Frequently, in an effort to develop interesting beer flavors, sugar sources other than barley malt are used (e.g., lentil [4], buckwheat [5], triticale [6], oats [7], black rice [8]). Another method of influencing organoleptic characteristics is through enriching beer with new, herb-derived aromas, as well as other aromas originating from biological materials [9]. A recent and very promising method of creating unique beer has been the application of non-*Saccharomyces* strains, known from the wine industry, in combination with brewing technology. This may result in beer with a desirable physiochemical composition and unique flavor profile. It should also be noted that non-*Saccharomyces* yeasts have been used in beer production from the onset as a component of spontaneous fermentation microbiota [10]. Through their metabolic, physiological or cellular functions, these microorganisms significantly change the organoleptic properties of food products [11].

The effect of many non-*Saccharomyces* yeast strains on the brewing process has been previously reported [12]. Most of the studied yeasts had low alcohol production efficiency (e.g., *Torulaspora delbrueckii* [13,14,15], *Brettanomyces bruxellensis, Pichia kluyverii* [16], *Wickerhamomyces anomalus* [17], *Lachancea fermentati* [18]) which made them a very good material for the production of low-alcohol or alcohol-free beer [12]. In order to increase the efficiency of the fermentation, they were also used as mixed cultures with *Saccharomyces*, obtaining promising results comparable with pure *Saccharomyces* [14]. Producing beer with an average alcohol content (ca. 4–6% v/v) through using pure non-*Saccharomyces* yeast requires the use of strains with the ability to ferment maltose (e.g., *Lachancea thermotolerans* [19], or *Zygotorulaspora florentina* [16], etc.).

*L. thermotolerans* is an innovative representative of non-*Saccharomyces* cultures. First known as *Kluyveromyces thermotolerans*, it was separated from the other species of *Kluyveromyces* due to significant metabolic and genetic differences [11,20]. The strain is very common, occurring in different environments both natural and artificial. Furthermore, it is recognized that *L. thermotolerans* strains produce lactic acid in large quantities during alcohol fermentation, with a capacity of up to 16 g/L of product [21,22], moreover improving the aroma of beer through inter alia, high ethyl butyrate and ethyl acetate production in both pure and mixed fermentations [17]. These distinctive properties are characteristic for the known *L. thermotolerans* yeast when compared with many other microorganisms [23,24]. On the other hand, significant lowering of beer pH by some Lt strains (e.g., 0.6, as a result of lactic acid production [14]), can limit their use at tailoring flavors during the production of various styles of beers.

The aim of the present study was to determine the low lactic acid production efficiency of the new *L. thermotolerans* MN477031 strain for brewing. Important parameters such as fermentation performance, resistance to hop compounds, sugar and nitrogen compound utilization and selected metal ions were reported. Furthermore, the new beer profile was compared to the reference trial (*S. cerevisiae*) by contrasting the interaction of the new yeast strain with different varieties of hop. Demonstrating the positive brewing properties of the yeast may prompt a wider application in the brewing industry of the *L. thermotolerans* MN477031.

## 2. Materials and Methods 

### 2.1. Materials

#### 2.1.1. Yeast Strains

The *L. thermotolerans* strain MN477031 yeast (Lt) was isolated from grape must in Slovakia and used for fermentation. It was previously identified and characterized by 2.6. 5.8S-ITS rRNA gene region sequencing (GenBank NCBI database accession number MN477031). The *S. cerevisiae* (Sc) yeast of the Safbrew T-58 strain (Fermentis Division of S.I.Lesaffre, Marcq-en-Baroeul, France) was used as a reference sample due to it having a fermentation temperature similar to Lt along with a high amount of spice and a fruity compound production.

#### 2.1.2. Hops

The Polish hop varieties were Lubelski (2.7% alpha acids) and Marynka (7.4% alpha acids) (Polish Hops, Karczmiska Pierwsze, Poland) were used.

### 2.2. Methods

#### 2.2.1. Wort Production

All-malt, first worts (18.6%) were produced by infusion mashing in a craft brewery from pilsner malt (Ireks, Kulmbach, Germany). After lautering, worts were standardized to a 12% extract with distilled water, and 250 mL of the wort was boiled in lab conditions for an hour with reflux condensers (to reduce vaporization). At the beginning of boiling, the Polish hops variety Lubelski (2.7% alpha acids) and Marynka (7.4% alpha acids) were dosed at 5 and 2 g/L, respectively. After boiling, the hot trub was removed from the worts; subsequently, the samples were cooled to 20 °C. Before inoculation, the key quality parameters of the worts were analyzed. The strongly significant differences between worts were only related to parameters of hop origin (Table 1). The values of obtained International Bitterness Unit (IBU) were 40 and 53 for Lubelski and Marynka hopped worts, respectively. The level of the extract, maltose (as the main wort sugar), glucose and free amino nitrogen (FAN) content were similar among the worts.

#### 2.2.2. Yeasts Propagation and Fermentation

All yeast strains were maintained on Sabouraud Dextrose LAB-AGAR (Biocorp, Warsaw, Poland) plates at 5 °C. Yeasts growing for 24 h on Sabouraud Dextrose LAB-AGAR at 25 °C were picked into a flask containing 20 mL of liquid Sabouraud broth (Biocorp, Warsaw, Poland) and grown at 25 °C for 2 days on a rotary shaker shaking at 121 rpm. After that the yeasts were centrifuged (735× *g*) for 15 min, washed with sterile distilled water, centrifuged again, and suspended in 10 mL of hopped wort (Marynka, Lubelski). The amount of cells in 1 mL of suspension was evaluated using the Thoma chamber (in triplicate).

Marynka (M (100 mL))- and Lubelski (L (100 mL))-hopped worts were inoculated with *L. thermotolerans* (Lt) or *S. cerevisiae* (Sc) yeast cell suspensions to obtain 7.65 × 10^6^ cells/mL, and fermented in rubber-stoppered Erlenmeyer flasks with fermentation tubes. The samples were fermented at 20 °C in a Q-CELL thermostatic chamber with daily measurements of weight loss. All activities were performed in sterile conditions.

#### 2.2.3. Analytical Determinations

Ethanol, real extract and apparent degree of fermentation (ADF) were measured by the automatic wort and beer analyzer (Alcolyzer, Anton Paar DMA 4500+). A sample of degassed beer was mixed with diatomaceous earth and filtered through a paper filter to obtain ~50 mL of filtrate. The filtrate was degassed for 20 min (universal shaker, 150 rpm), adjusted to 20 °C and filtered again.

The pH of the beers was measured using the Mettler Toledo FiveGo pH-meter.

Volatile compound analysis (SPME-GC-MS): In order to determine the headspace volatile compounds, 1 g of NaCl and a 2-mL sample of wort/beer were placed into a 10-mL vial. Next, an internal standard solution was added (0.57 mg/L 4-methyl-2-pentanol, 0.2 mg/L anethol and 1.48 mg/L ethyl nonanoate, Sigma-Aldrich, St. Louis, MO, USA). The SPME device (Supelco Inc., Bellefonte, PA, USA) coated with PDMS (100 μm) fiber was first conditioned by inserting it into the gas chromatograph injector port at 250 °C for 1 h. For sampling, the fiber was inserted into the headspace under stirring (300 rpm) for 30 min at 60 °C. Subsequently, the SPME device was introduced into the injector port of the Agilent Technologies 7890B chromatograph system equipped with LECO Pegasus High Throughput TOFMS, and kept in the inlet for 3 min. The SPME process was automated using the GERSTEL MultiPurpose Sampler (MPS). The tested components were separated on a Rtx-1ms capillary column (Crossbond 100% dimethyl polysiloxane, 30 m × 0.53 mm × 0.5 μm). The detector was 250 °C, and the column was heated using the following temperature program: 40 °C for 3 min at an increment of 8 °C/min to 230 °C, then maintaining a constant temperature for 9 min. Carrier: Helium at 1.0 mL/min constant flow. EIMS electron energy 70 eV; ion source temperature and connection parts: 250 °C. The analyte transfer was performed in splitless mode; the MSD was set to scan mode from m/z = 40 to m/z = 400. Compounds were identified using mass spectral libraries and linear retention indices, calculated based on a series of n-alkanes from C6 to C30. The qualitative and quantitative identification of volatile substances (ethyl acetate, ethyl butanoate, isoamyl acetate, ethyl hexanoate, ethyl octanoate, 2-phenylethyl acetate, ethyl decanoate, ethyl dodecanoate, ethyl tetradecanoate, ethyl hexadecanoate, ethyl octadecanoate, 2-methyl-1-propanol, 2-methyl-1-butanol, 2,3-butanodiol, 1-hexanol, 3-methyl-1-butanol, 2-phenylethanol, 1-nonanol, 1-decanol, hexanoic acid, octanoic acid, n-decanoic acid, limonene, linalol oxide, linalool, α-terpineol, citronellol, geraniol, ß-damascenone, ß-caryophyllene, humulene, caryophyllene oxide, decanal; Sigma-Aldrich) were based on a comparison of retention times and peak surface area reads from samples and standard chromatograms. Other detected components were determined semi-quantitatively (µg/L) by measuring the relative peak area of each identified compound according to the National Institute of Standards and Technology (NIST) database [25] in relation to that of the internal standard. Each of the tests were performed three times.

Organic acid analysis: High performance liquid chromatography was applied for the analysis of the organic acids and the sugar profile. An HPLC analysis was carried out on a Perkin-Elmer (USA) FLEXAR chromatograph with a UV-Vis detector. Malic, oxalic, succinic, lactic, citric and acetic acids (Sigma-Aldrich) were determined using the Rezex ROA-Organic Acid Aminex HPX-87H (300 mm, 18 cm × 7.8 mm). Samples were eluted isocratically at 40 °C with a mobile phase (0.005 M H_2_SO_4_) at a flow rate of 0.4 mL/min [26].

Content and profile of sugars: The content of sugars was determined using the HPLC method. Analysis of the sugar profile was conducted with the Shimadzu (Kyoto, Japan) NEXERA XR apparatus with a RF-20A refractometric detector. The separation was conducted with an Asahipak NH2P-50 4.6 × 250 mm Shodex column (Showa Denko Europe, Munich, Germany) thermostated at 30 °C. The mobile phase consisted of an acetonitrile aqueous solution (70%), and the isocratic elution program (0.8 mL/min) lasted 16 min.

Quantitative determinations were made with the use of standard curves prepared for the appropriate standards: glucose, maltose and glycerol. The tables of results only present the concentrations of sugars with amounts higher than the method’s detection threshold.

Weight loss during fermentation, related to release of CO_2,_ was measured by the RADWAG WPS 600/C scale.

Free amino nitrogen (FAN) was measured using ninhydrin-based methods with the use of the absorbance measurement at 450 nm (Beckman DU-650 UV-Vis) according to the Analytica EBC [27] method 8.10_Free Amino Nitrogen in wort by Spectrophotometry (IM).

The International Bitterness Unit (IBU) was measured with the use of isooctane extraction of iso- α-acids from acidified samples. After extraction, the absorbance analysis was conducted (Beckman DU-650 UV-Vis) with the use of a 275-nm wavelength according to Analytica EBC method 8.8_Bitterness of wort, the determination of the bitter substances, mainly iso- α-acids, in wort.

The color of the filtered beer was measured spectrophotometrically (Beckman DU-650 UV-Vis) at a wavelength of 570 nm according to Analytica EBC method 8.5_Colour of wort: Spectrophotometric Method (IM).

Ion concentration: Before analyzing metal ions, wort and beer samples were mineralized to achieve the complete decomposition of their organic substances. Next, 2 mL of each sample was added to mineralizing dishes, and HNO_3_ was poured over the samples (65%, 3 mL). The samples were wet mineralized in a Mars Express microwave oven (at a maximum temperature of 170 °C, duration: 40 min). After wet mineralization, the contents of the mineralization dishes were transferred in quantity to test tubes and supplemented with deionized water to reach 14 mL. Magnesium, calcium and zinc ions were analyzed with the VARIAN 240FS spectrometer using an atom spectrometer absorption method with flame atomization (air/acetylene). The device uses the automatic sample dosing system SPIS-20. Mg^2+^ ion absorbency was determined at a wavelength of 202.6 nm, Ca^2+^—422.7 nm, Zn^2+^—213.9 nm. A fast sequential mode was run for the determination during a single aspiration of a sample.

#### 2.2.4. Statistical Analyzis

The results are presented as the average of three or more independent experiments with the bars representing the standard deviation. The data were analyzed through a one-way analysis of variance (ANOVA). The significance in the difference for each parameter was analyzed separately, using Tukey’s range test (Statistica v. 10, StatSoft Inc., Krakow, Poland).

## 3. Results

### 3.1. Fermentation Performance

**Figure 1:**
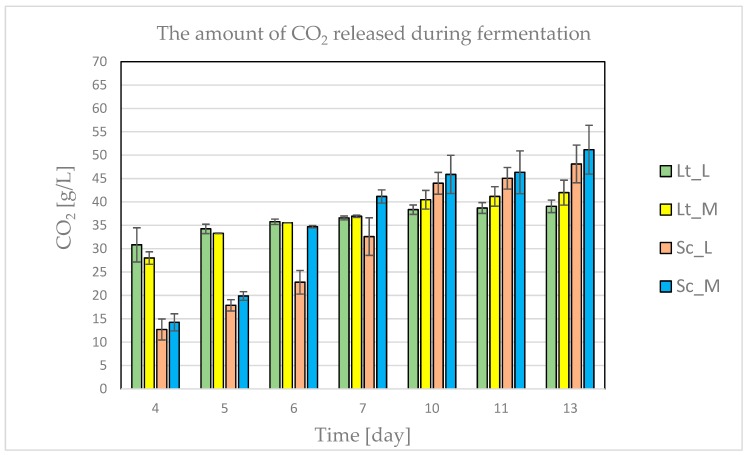
Carbon dioxide evolved during wort (hopped with Marynka (M) or Lubelski (L)) fermentation with *L. thermotolerans* (Lt) or *S. cerevisiae* (Sc) yeast.

Wort fermentation progress was monitored by measuring the carbon dioxide release (g/L) over the following days of the process: From the time of wort inoculation to the fourth day of the process, samples inoculated with Lt produced twice the amount of carbon dioxide compared to samples inoculated with Sc (Figure 1). This may have been caused by the higher adaptability of the Lt strain to the fermentation conditions. Furthermore, the rate of the Lt process was not affected by the hop variety. During Sc fermentation, differences between fermentation media were observed only on the sixth and seventh days of the process. The results are similar to those obtained by Domizio et al. [19], where the progress of the process was not affected by the IBU level either at 30 or 60 IBU. Bellut et al. [18] also observed the minor impact of a high IBU value on the *L. fermentati* KBI 12.1. The volume of CO_2_ released between days six and seven of the process for all samples began to stabilize. However, from day 10 to the end of the process, higher volumes of evolved CO_2_ were found in the Sc samples.

The high intensity of fermentation during the first days of the process is rarely observable in literature in the case of *L. thermotolerans* strains. However, the volume of CO_2_ at the later stages of the process presented a level similar to the results of Porter et al. [28], who analyzed *L. thermotolerans* (*Concerto* and *Y940*) and *S. cerevisiae* yeast fermentation kinetics. It was proven that, regardless of the fermentation medium used (synthetic grape juice or natural Muscat juice), the process rate was lower in the case of non-*Sacharomyces* monocultures. The differences in CO_2_ release reached approximately 27% less for Concerto and 36% less for Y940. Similarly, Gobbi et al. [29], during the 24-day fermentation process of white grape, must have found a reduced rate of *L. thermotolerans* 101 culture fermentations compared to the pure culture of *S. cerevisiae* or *S. cerevisiae* with *L. thermotolerans*. According to Benito [22], *Lachancea* strains are characterized by relatively good fermentability; however, due to the longer transformation of sugars into alcohol, and the relatively high quantity of unfermented sugars left in the final product, the yeasts debuted in fermentations mixed with *S. cerevisiae*. Such combinations solve the above-mentioned issues and allow for considerably faster and more effective alcohol beverage production. Studies conducted by Balikci et al. [30] confirmed that the fastest reduction (from nine to seven days) of the original gravity was led by *S. cerevisiae,* as well as by the mixed *S. cerevisiae* and *L. thermotolerans* cultures in different variants against a single *L. thermotolerans* or variants initially inoculated with *L. thermotolerans*, followed by *S. cerevisiae.* Following analyses of various non-*Saccharomyces* yeasts, Plessis et al. [31] found two *L. thermotolerans* strains with fermentation capacities comparable to *S. cerevisiae.* This could be an indication of the reason why *L. thermotolerans*—as with *T. delbrueckii*—are selected from their species for commercial mixed cultures with *S. cerevisiae*. Interesting data were acquired by Comitini et al. [32] in the comparison of 34 non-*Saccharomyces* strains to some selected *S. cerevisiae* strains: he demonstrated that the mean daily CO_2_ produced by *L. thermotolerans* ranged from 0.03 g/L to approximately 1.5 g/L, depending on the strain, while in the case of *S. cerevisiae,* the range would start at 1.3 and exceed 2.5 g/L. In the Canonico et al. [17] study, the total CO_2_ released after 20 days of fermentation conducted by different strains of *L. thermotolerans* also varied (between 20 and 39 g/L) in comparison with 46 g/L evolved from wort by *S. cerevisiae*. This proves that, when comparing fermentation kinetics between *S. cerevisiae* and *L. thermotolerans*, the key role is a specific strain. In studies by Benito et al. [33], process kinetics were presented as having the capacity to utilize glucose and fructose from a fermentation medium over time. In this case, differences in the duration of the process ranged from 8 to 16 days, depending on the application of pure *S. cerevisiae* or a combination of *L. thermotolerans* and *S. pombe* strains.

### 3.2. Beer

After 14 days of fermentation, the alcohol content in the beers ranged from 4.26% to 5.37% (v/v), while the real extract ranged from 3.92% to 5.46% (w/w) (Table 2). The highest volume of ethanol was determined in beers produced with the use of Sc yeast: 5.37% in beer made with Lubelski hop and 5.22% in beer with Marynka. The percentage of ethanol content in beers fermented using Lt was significantly lower (4.3–4.25%), irrespective of the type of hops used. This is reflected in the real extract content; its highest value was found in Lt samples, in contrast with the lowest ADF found in the strain (67%). This may indicate a lower capacity for alcohol production by the analyzed strain when compared to Sc yeast. Holt et al. [16] tested the fermentation efficiency among 23 non-*Saccharomyces* yeast strains. *Z. florentina* and *L. thermotolerans* were the most effective at producing 4% and 2.6% v/v alcohol, respectively. The average alcohol content for all non-*Saccharomyces* yeast strains was 1.4% v/v.

These findings are consistent with results of other authors, starting with Kapsopoulou et al. [34], who were among the first to observe the dependencies, achieving an ethanol concentration at 7.58% (v/v) and 9.6% (v/v) in wines as a result of fermenting *L. thermotolerans* and *S. cerevisiae.* Subsequent research [28,35] confirmed this thesis, demonstrating increasing differences between *L. thermotolerans* and *S. cerevisiae*. According to the works, *L. thermotolerans* generated 8.3% (v/v) and 8.9% (v/v) of alcohol content against 11.1% (v/v) and 12.3% (v/v) of ethanol in the case of *S. cerevisiae,* which is similar to the findings of Comitini et al. [32] which were 4.58–7.96% in comparison to 10.78–12.62%, respectively. A lower alcohol production capacity demonstrated by *L. thermotolerans* strains as compared to *S. cerevisiae* strains does not signify the incapability of the yeast to biosynthesize increased volumes of the compound. By providing adequate conditions during the process, several authors succeeded in exceeding the 10% (v/v). Du Plessis et al. [31] obtained 10.35% (v/v), Gobbi et al. [29] obtained 10.46% (v/v), Hranilovic et al. [11] obtained 10.6% (v/v) and Balikci et al. [30] obtained 10.91% (v/v) of ethanol. Nevertheless, it should be noted that, in the control samples (*S. cerevisiae*) of the above, the alcohol content was at least equal or higher. From the point of view of using *L. thermotolerans* in the brewing industry, resistance to a high concentration of alcohol is not the key criterion. Their potential for beer production was demonstrated by Domizio et al. [19]. In the process of fermenting wort and applying *L. thermotolerans* when compared to *S. cerevisiae* strains, the final alcohol content obtained in the test was only marginally lower (from 6% to 12%) than in the strains used in the brewing industry. Canonico et al. [17] noted higher differences in ethanol concentrations between pure cultures of tested strains (ca. 22%), while in the case of mixed fermentations, the obtained results were not significantly different. It is reasonable to assume that the lower capacity for transforming sugars to ethanol—demonstrated by *L. thermotolerans*—may be a positive factor when producing fermented beverages such as beer or sparkling or sweet wine with reduced alcohol content [33,36,37]. Furthermore, in beers produced with *L. thermotolerans* strains, a higher content of real extract was observed, which is justified by the lower ethanol content produced in the process. This may indicate that the available sugars are used to a lower degree by yeasts in ethanol biosynthesis, and that sugars can be used to generate higher amounts of other compounds (e.g., glycerol—a beer extract component).

### 3.3. Sugars and Organic Acids

Before fermentation, worts were characterized by similar levels of maltose (of approximately 85 g/L) and glucose (13 g/L) (Table 1). Following the fermentation process, the observation was made that the Lt strain had fully consumed the glucose when compared to Sc at 0.040 and 0.036 g/L. In the case of maltose, its consumption was similar and—solely in the case of Sc beer hopped with the Marynka variation—slightly more maltose was left. This observation is reflected in the results of Domizio et al. [19], where *L. thermotolerans* and *S. cerevisiae* strains demonstrated similar maltose fermentation capacities, obtaining approximately 93–94% of maltose consumption. Callejo et al. [38] proved a low maltose fermentation capacity by the *L. thermotolerans* kt 421 strain compared to the strong fermentation by the *S. cerevisiae* 7VA strain. Interesting results were obtained by Canonico et al. [13]: among the 28 non-*Saccharomyces* stains (*T. delbrueckii*), only eight fermented maltose, whereas all of them had the ability to ferment glucose and sucrose. None of the strains showed comparable fermentation parameters to *S. cerevisiae* US-05, so they are suitable for the production of low-alcohol beers. One of the strains (*T. delbrueckii* DiSVA 254) which had the highest fermentation rate and CO_2_ production was used for further tests both as a pure culture and mixed with *S. cerevisiae* US-05. However, when analyzing the fermentation capacities of strains, the observations of Porter et al. [28] should also be taken into consideration. These indicated that the capacity was not only strain-dependent but also dependent on the fermentation medium. In synthetic grape juice, after the fermentation of *S. cerevisiae*, approximately 1 g/L of glucose and approximately 2.5 g/L of fructose were left compared to the same Muscat grape fermentation process, where neither glucose nor fructose were detected after the process. In the same study, from 10 to 45 g/L of glucose and approximately 37 to 57 g/L of fructose was left after synthetic juice fermentation, depending on the *L. thermotolerans* strain. Nevertheless, from 7 to 12 g/L of glucose and 22–28 g/L of fructose was left after Muscat juice fermentation. Significantly lower levels of residual sugars were reported by Gobbi et al. [29], with *S. cerevisiae* at approximately 6 g/L and *L. thermotolerans* at 55 g/L after fermentation. In the case of the research work by Balikci et al. [30], a comparison of wines produced by *S. cerevisiae* and *L. thermotolerans*, led to the conclusion that *S. cerevisiae* yeasts demonstrated higher consumption of both glucose and fructose, remaining in wine at approximately 0.8 and 1.3 g/L, respectively, which is contrary to the higher content of these compounds in pure *L. thermotolerans* wine (2.3 and 4 g/L, respectively). Research carried out by Canonico et al. [17] also proved the lower fermentation ability of non-*Saccharomyces* yeast strains. The final gravity of beer produced by pure *L. thermotolerans* was higher (1.023) than that of *S. cerevisiae* (1.01).

No lactic, oxalic, acetic, citric, malic or succinic acids were found in the worts. An insignificant difference in the biosynthesis of organic acids was found in the finished beer (Table 3). A small concentration of lactic acid in the analyzed strain contradicts tests on other *L. thermotolerans* strains, which evidenced their higher levels. Domizio et al. [19] even described the *L. thermotolerans* 101 strain as a micro-organism representing a good alternative for sour beer production. The absence of a similar observation prevents the boarder application of the MN477031 strain in brewing across different styles of beer. Furthermore, only marginal differences in the pH of Lt and Sc beers signify the large potential of the analyzed strain.

### 3.4. Glycerol

No glycerol content was observed in the worts, while its concentration in the finished product was reported at approximately 4.5 g/L for Sc, and approximately 9 g/L for Lt (Table 2). In many studies, a similar trend of *L. thermotolerans* producing an increased amount of glycerol to reach approximately 8.0 g/L was observed [11]. A percentage increase in the volume of the metabolite after 21 days of the fermentation was estimated to range from 65% to 75% [19]. Similar to the case of ethanol in mixed fermentations, glycerine concentrations are dependent on the moment of introducing the *S. cerevisiae* culture. These differences may reach up to 1.12 g/L. The final glycerol content is also influenced by the temperature. Fermentation at 30 °C results in an increase in the glycerol produced in the process when compared to fermentation at 20 °C [22]. Similarly, tests by Shekhawat et al. [39] proved that increased aeration during fermentation, both of pure *L. thermotolerans* cultures as well as those mixed with *S. cerevisiae,* leads to increased glycerol production with a simultaneous drop in ethanol efficiency. This was also seen and reflected in our study in the increased real extract in the case of Lt (Table 2), which includes glycerol [40]. In the reference fermentations (Sc), the amount of glycerol obtained was very similar to the control culture of Kapsopoulou et al. [41], which was *S. cerevisiae.*

### 3.5. FAN

Nitrogen compounds play an important role in the production of alcoholic beverages, mainly through their assimilation or metabolism by yeast cells during ethanol fermentation. In the brewing industry, they are very often referred to as FAN (free amino nitrogen) and contain individual alpha amino acids, ammonia and small peptides. In the wine industry, together with ammonium salts they are called YAN (yeast assimilable nitrogen). They can be also referred to as PAN (primary amino nitrogen), total usable nitrogen or usable nitrogen [42,43,44]. The FAN content in worts results from the barley malting process [45], mashing temperature profile [46,47] and content of nitrogen compounds in barley. Tests by Psota et al. [48] demonstrated that the FAN content in sweet wort and grains is largely dependent on the year of barley growing (20%), the place of its growing (14%) and its variety (13%). Literature offers different recommended FAN levels for brewing wort [43]. In the case of Lt and Sc fermented samples, the obtained 130 to 140 mg/L is consistent with the minimum recommendations by Stewart et al. [49] and Butzke [50] for worts with 10–12°P extract. What is important for this paper is that these requirements [43] have been met both for the brewer’s and wine yeasts (Table 2). The lack of FAN in wort could cause slowed fermentation and poorer diacetyl reduction. In addition, FAN deficits are often accompanied by deficits of other nutrients and—in such cases—the deficit of absorbed nitrogen cannot be compensated by mineral or lipid supplementation [42]. On the other hand, an excessive level of FAN is not recommended, either. The Brewers Association recommends maintaining FAN at the necessary, minimum level [51]. During the fermentation process, a large amount of FAN is used by yeasts for building protein - supporting biomass growth. In addition, it is partially used for creating volatile compounds, including higher alcohols, esters, diacetyl and H_2_S [52]. FAN content in ready beer is dependent on many factors and its value may widely vary. Its levels found in beers produced using Sc and Lt yeasts did not show any statistically significant difference in terms of the variety of hops used. These oscillated around 41–53 mg/L (Sc) and 57–66 mg/L (Lt), which is similar to the findings of Jaskula-Goris et al. [53] in the case of the lowest value for fresh beer 58.8 mg/L, and is significantly different from its highest concentration of 124.1 mg/L. In tests performed by Bellut et al. [18], *S. cerevisiae* demonstrated the capacity to utilize approximately 60% of FAN against the 65% recorded in this research. On the other hand, the analyzed *L. fermentati* KBI 12.1 strain used FAN at no more than 30% when compared to 55% for the Lt strain reported in our research. An excessive volume of FAN in beer is not recommended, largely owing to its potential to contribute to the generation of undesirable products (e.g., Mailard’s reactions or Strecker degradation [53]), and thus potentially reducing beer stability [54].

### 3.6. Esters, Alcohols and Other Volatile Compounds

One of the key objectives for using non-*Saccharomyces* yeast in brewing is the modification of the sensory profile of beverages. Generally, such methods are recognized as the concept of bioflavoring [52]. The main compounds that influence the aroma of alcoholic beverages are esters, higher alcohols, terpenes, acids and so on [55]. In addition, ethanol is also an important factor in the organoleptic perception of beer. As a result of the fermentation, many new volatiles have appeared in beer, and some of them have significantly increased their concentration (Table 4). Furthermore, the analysis of volatile compounds leads to the observation that beers produced using Lt demonstrated a significant difference from the beer brewed with Sc. In order to prove the differences in the concentrations of volatile compounds, a decision was made to first prove that the differences were occurring at the same time, irrespective of the variety of hops used. In case of ester compounds in beer, Lt application significantly reduced the concentration of almost all the analyzed esters (Table 4). The exceptions were ethyl 2-methyloctanoate (higher concentration in Lt) and ethyl acetate, whose concentration in the Sc_M sample was comparable to that of Lt. The obtained results are in agreement with other studies (e.g., Balikci et al. [30]), proving the lower amount of ester in *L. thermotolerans* products. Canonico et al. [17] obtained a higher concentration of ethyl butyrate and ethyl acetate in beer produced by *L. thermotolerans* and, similar to the present study, a lower concentration of other measured esters (2-phenylethyl acetate, ethyl hexadecanoate and isoamyl acetate). In the case of several ester compounds, the variety of hops used in the process was also important for Sc beers (e.g., in case of ethyl 9-decenoate). The difference in ester concentration between produced beers is very important to overall sensory expression. This is mainly due to their low thresholds and positive effect on the aroma of beers, introducing floral and fruity notes. Higher glucose and fructose content in wort leads to higher concentrations of ester than in maltose-rich worts [38,56]. In Lt-fermented beers, the content of alcohols 3-ethoxy-1-propanol, 1-nonanol and 1-decanol were significantly higher than in Sc, whereas the 2-methyl-1-propanol, 2-methyl-1-butanol, 2,3-butanediol, 3-methyl-1-butanol and 1-heptanol were formed in lower concentrations compared to in the reference sample. In the study by Callejlo et al. [38], similar trends were observed for 2-methyl-1-butanol and 3-methyl-1-butanol. Gobbi et al. [29] and Canonico et al. [17] also reported a significant reduction of 2-methyl-1-propanol and 3-methyl-1-butanol levels when producing wine or beer with a pure *L. thermotolerans* strain as compared with *S. cerevisiae*. In research by Balikci et al. [30], a sample obtained by a pure culture of *L. thermotolerans* was also characterized by a lower concentration of 2-methyl-1-propanol, 2-methyl-1-butanol and 3-methyl-1-butanol and a more than twofold increase in the concentration of N-propanol. For most of the volatile organic acids detected, smaller amounts were also found in beers obtained with the *L. thermotolerans* yeasts. In terms of acids, only in hexanoic and octanoic acid was the lower amount statistically significant. This may explain why the samples contained smaller amounts of esters for which the acids were precursors [57,58]. No significant differences dependent on application of Lt were observed in cases of phenols.

Decanal (sweet, waxy, orange, citrus peel aroma) and 2-dodecanol (floral aroma)—representing carbonyl compounds—were found at a significantly higher quantity in Lt when compared to its presence in Sc. It has been proven in other research that it is not only the selection of the strain and fermentation medium that have a significant impact on the volatiles profile, but also the ratio of individual microorganisms in the mixed culture. Morales et al. [35] presented the content of volatile compounds depending on the strain dominant during inoculation; in this manner, the dominant *L. thermotolerans* culture in the environment triggered an increase in alcohol levels, while *S. cerevisiae* caused a linear reduction in acetals, acids and ethyl esters accompanied by its increased share during inoculation. Comitini et al. [32] demonstrated that mixed cultures of *L. thermotolerans* intensified the production of 2-phenylethanol and reduction in octanoic acid. Benito et al. [33] determined an increased level of higher alcohols in samples engaging *L. thermotolerans* and *S. cerevisiae* when compared to *S. pombe* samples.

### 3.7. Terpenes

Terpenes are the main constituents of hops’ essential oils and contribute to the singular sensory properties of beer. More than 90% of the hop oil volatilizes during the boiling period and the traces are found in the hopped wort [59]. Although β-myrcene, α-humulene, β-caryophyllene and β-farnesene have been shown to be the main components of hop oil, the predominant components in finished beer are terpene alcohols, especially linalool and geraniol [60]. Different yeast species have different ability to release terpenes from the bound form (β-glicosidase activity) and metabolize them to other terpenes [61]. However, there is not much information in the literature about such transformations of non-*Saccharomyces* yeasts, which significantly differ in metabolism from starter brewing cultures. Worts hopped with two different hops (Marynka, Lubelski) were characterized by a similar composition of terpenes, among which geraniol, humulene epoxide II, humulene, β-farnesene and patchulane dominated (Table 5). After fermentation, significant changes in the quantitative and qualitative composition of the terpenes were observed, with more of these compounds found in beers obtained with *L. thermotolerans* yeasts. Limonene, perillen, nerol and humulene were completely used in all samples, as well as perilla alcohol and caryophyllene oxide in samples fermented by *S. cerevisiae*. At the same time citronellol, β-damascenone and nerolidol were formed, terpenes were not detected in unfermented worts. In Lt beers, terpenes derived from hops—such as humulene epoxide II, geraniol and patchulane—predominated. To a lesser extent, those produced de novo (e.g., citronellol and linalool) were, in contrast to Sc beer dominated by citronellol, humulene epoxide II, patchulane and linalool. The above tendency is very important for the production of beers with a modified, specific aroma in which the presence of terpenes can be significantly more perceptible due to the smaller amounts of other aroma compounds such as esters and alcohols. In the research of Canonico et al. [17] on terpene concentrations in beer, the citronellol amount was significantly lower in beer produced by pure *L. thermotolerans* in comparison with beer fermented by pure *S. cerevisiae* or mixed with *L. thermotolerans*.

### 3.8. Metal Ions

An important factor affecting the fermentation process and beer quality is the ion composition of the worts. Some ions (e.g., Mg^2+^ and Zn^2+^) have a significant impact on the vitality of yeasts and support the progress of the fermentation process [62]. The content of trace quantities of the elements in raw materials and final products of the brewing industry should be under continuous control. The controlling amount of ions in raw materials and process operations impact the ion content in the wort significantly [63]. Brewing yeasts demonstrates a high demand for magnesium ions in the wort (ranging from 50 to 150 mg/L or more), which is rather uncommon for other metals [63]. A deficit of this element causes a number of complex and often subtle changes in yeast cells physiology [63,64]. In pitching worts, the content of magnesium reached nearly 200 mg/L (Table 6) and, for this reason, wort supplementation was not necessary in order to improve the fermentation process. Calcium was the next ion analyzed in the research. The optimum Ca^2+^ content for the proper growth of the yeasts and progress of the fermentation process was determined in the range of 30–60 mg/L of wort [63]. All the analyzed samples had the appropriate calcium content, consistent with the data presented in the literature. Zinc is required for the proper growth of yeasts and fermentation, although its content is not generally high. This element also contributes to the accumulation of sugars, and the optimum demand of yeast for zinc ranges from 0.1 to 1 mg/L of wort [65,66]. The analyzed samples were characterized by a higher content of the element. This must have been caused by the high zinc extraction from malt during mashing and mash filtration.

## 4. Conclusions

This study examined the impact of the *L. thermotolerans* MN477031 strain on brewing production. The progress of the fermentation process was monitored for two types of worts. It was investigated that the MN477031 strain had a high adaptability to the fermentation conditions and produced more than 4% v/v ethanol from the 12% w/w wort, which is above average for non-*Saccharomyces* yeast strains. Furthermore, the aroma profile of beer was changed due to, for example, lower esters and volatile acids concertation compared to the Safbrew T-58 strain.

One of the key characteristics of the *L. thermotolerans* MN477031 strain was the low lactic acid production and only a minor impact on the reduction in pH of the beer, in comparison with the reference sample and known *L. thermotolerans* strains.

In addition, the following characteristics provide high potential for the application of the MN477031 strain in different beer styles: high maltose fermentation ability, resistance to higher IBU levels of a minimum up to 53, a similar requirement for FAN and metal ions in worts and higher glycerol productions in comparison to the Safbrew T-58 strain, which could improve the mouthfeel of the produced beers.

## Figures and Tables

**Table 1:** The composition of worts before fermentation, depending on the hop variety used.

	Extract	Glucose	Maltose	FAN	IBU
	% (w/w)	g/L		mg/L	
**Lubelski**	12.0 ± 0.02	12.52 ± 0.75	79.40 ± 4.71	131.07 ± 0.92	40 ± 2 ^a^
**Marynka**	12.0 ± 0.03	14.49 ± 2.58	83.03 ± 6.29	139.18 ± 0.77	53 ± 3 ^b^
**Sig. ^1^**	ns	ns	ns	ns	***

^1^ Sig.: significance; *** display the significance at 0.5% by least significant difference; ns: not significant. Values with different superscript roman letters (a–b) in the same column are significantly different according to the Tukey’s range test (*p* < 0.05). International Bitterness Unit (IBU); and free amino nitrogen (FAN).

**Table 2:** Physicochemical parameters of beers depending on the strains and hop variety.

	*L. thermotolerans* MN477031		*S. cerevisiae* Safbrew T-58		Sig. ^1^
	Lubelski	Marynka	Lubelski	Marynka	
**Ethanol (% v/v)**	4.3 ± 0.02 ^a^	4.25 ± 0.17 ^a^	5.37 ± 0.08 ^b^	5.22 ±0.16 ^b^	***
**Real extract (% w/w)**	5.46 ± 0.017 ^a^	5.41 ± 0.010 ^a^	4.03 ± 0.035 ^b^	3.92 ± 0.028 ^c^	***
**Apparent Degree of Fermentation (%)**	67.38 ± 0.1 ^a^	67.31 ± 1.2 ^a^	82.83 ± 0.28 ^b^	82.83 ± 0.69 ^b^	***
**pH**	4.43 ± 0.1 ^a^	4.39 ± 0.09 ^a^	4.72 ± 0.08 ^b^	4.67 ± 0.11 ^b^	**
**Color (EBC)**	8.0 ± 0.91 ^a^	6.9 ± 0.36 ^a^	14.0 ± 0.36 ^b^	7.5 ± 0.12 ^a^	***
**Glycerol (g/L)**	8.61 ± 2.33 ^a^	9.29 ± 2.37 ^a^	4.72 ± 1.14 ^b^	4.48 ± 1.25 ^b^	**
**FAN (mg/L)**	56.6 ± 9.7	65.9 ± 6.5	41.32 ± 14.1	53.38 ± 2.8	ns

^1^ Sig.: significance; ** and *** display the significance at 1% and 0.5% by least significant difference; ns: not significant. Values with different superscript roman letters (a-c) in the same row are significantly different according to the Tukey’s range test (*p* < 0.05).

**Table 3:** Concentration of glucose, maltose and organic acids in beers hopped with Marynka (M) or Lubelski (L) hop pellets fermented by *L. thermotolerans* (Lt) or *S. cerevisiae* (Sc).

	Glucose	Maltose	Oxalic Acid	Acetic Acid	Citric Acid	Malic Acid	Succinic Acid	Lactic Acid
	g/L							
**Lt_L**	0.00 ± 0.00 ^a^	2.786 ± 1.99 ^a^	0.082 ± 0.03	0.160 ± 0.03	0.000 ± 0.00	0.030 ± 0.01	0.170 ± 0.1	0.01 ± 0.00
**Lt_M**	0.00 ± 0.00 ^a^	2.811 ± 2.74 ^a^	0.075 ± 0.02	0.174 ± 0.13	0.003 ± 0.00	0.052 ± 0.02	0.275 ± 0.08	0.06 ± 0.07
**Sc_L**	0.039 ± 0.02 ^b^	3.628 ± 1.17 ^a^	0.031 ± 0.01	0.390 ± 0.04	0.116 ± 0.00	0.039 ± 0.01	0.324 ± 0.04	0.00 ± 0.00
**Sc_M**	0.036 ± 0.01 ^c^	4.034 ± 0.04 ^b^	0.039 ± 0.04	0.232 ± 0.18	0.045 ± 0.00	0.025 ± 0.01	0.233 ± 0.2	0.00 ± 0.00
**Sig. ^1^**	**	*	ns	ns	ns	ns	ns	ns

^1^ Sig.: significance; * and ** display the significance at 5% and 1% by least significant difference; ns: not significant. Values with different superscript roman letters (a–c) in the same column are significantly different according to the Tukey’s range test (*p* < 0.05).

**Table 4:** Volatile compounds in beer brewed with Marynka (M) or Lubelski (L) hop pellets, fermented by *L. thermotolerans* (Lt) or *S. cerevisiae* (Sc).

Compound [µg/L]	LRI ^2^	Lt		Sc		Sig. ^1^
		L	M	L	M	
**Esters**						
Ethyl Acetate	614	4302 a	3200 a	8525 b	3116 a	***
n-Propyl acetate ^3^	694	0 b	0 b	76.6 b	21.5 a	**
Ethyl butanoate	789	7.4 a	8.1a	34.8 b	36.2 b	***
Isoamyl acetate	872	43.0 a	65.0 b	271.7 c	293.8 c	***
Ethyl hexanoate	986	4.1 a	8.6 a	116.4 b	91.1 b	***
Ethyl octanoate	1180	10.4 a	11.8 a	1776.6 b	2480.4 b	***
Ethyl 2-methyloctanoate ^3^	1209	69.7 a	43.2 a	10.7 b	29.2 b	***
2-Phenylethyl acetate	1228	55.8 a	57.8 a	777.14 b	2863.32 c	***
Ethyl 9-decenoate ^3^	1389	0.6 a	0.4 a	38.6 b	105.9 c	***
Ethyl decanoate	1397	4.4 a	2.7 a	306.2 b	392.9 b	***
Isobutyl decanoate ^3^	1546	0 a	0 a	0.24 b	0.60 c	***
Ethyl dodecanoate	1581	0.8 a	0.4 a	48.3 b	38.1b	***
Benzyl Benzoate ^3^	1750	1.05	0.45	0.38	0.14	ns
Ethyl tetradecanoate	1790	1.1 a	0.4 a	4.8 b	3.2 b	***
Ethyl pentadecanoate ^3^	1880	0	0	0.6	0.8	ns
Ethyl 9-hexadecenoate ^3^	1977	1.04 ab	0.55 b	1.35 a	1.08 ab	***
Ethyl hexadecanoate	1990	3.4 a	1.5 a	10.9 b	9.8 b	***
Ethyl octadecanoate	2189	0.8	0.1	0.7	0.6	ns
**Alcohols**						
2-Methyl-1-propanol	617	123.2 a	113.7 a	343.8 b	441.4 b	***
3-Methyl-1-butanol	723	8856 a	7379 a	11105 b	17997 c	***
2-Methyl-1-butanol	740	2413 a	1792 a	3888 b	6544 c	***
2,3-Butanediol	768	174 a	136 a	585 b	1446 c	***
3-Ethoxy-1-propanol ^3^	862	276.5 a	91.7 a	0 b	0 b	***
1-Hexanol	865	32.5 a	27.9 ac	26.9 ac	23.4 c	*
1-Heptanol ^3^	954	6.6 a	5.5 a	12.3b	12.8 b	**
2-Ethyl-1-hexanol ^3^	1020	37.8	31.7	23.6	24.9	ns
2-Phenylethanol	1084	2151 a	3807 b	3892 b	9196 c	***
1-Nonanol	1156	61.9 a	59.2 a	15.6 b	16.2 b	**
2-Propyl-1-heptanol ^3^	1203	16.3	15.8	11.3	10.9	ns
2-[(2-Ethylhexyl)oxy]-ethanol ^3^	1226	507 a	487 ab	481 ab	311 b	*
1-Decanol	1272	465 a	467.8 a	320.5 b	186.9 c	***
1-Undecanol	1374	37.0	33.1	20.4	9.8	ns
2-Dodecanol	1417	4.8	4.5	1.3	1.4	ns
**Acids**						
3-Methylbutanoic acid ^3^	833	112.2	75.9	116.2	139.6	ns
2-Methylbutanoic acid ^3^	858	16.9	0	31.5	21.8	ns
Hexanoic acid	982	16.5 a	11.9 a	44.0 b	79.9 b	***
Octanoic acid	1160	297 a	293 a	1724 b	2874 c	***
9-Decenoic acid ^3^	1358	11.0 a	4.8 b	12.3 a	18.8 c	*
n-Decanoic acid	1368	69.3 a	46.6 b	72.9 a	126.5 c	***
**Phenols**						
2-Methoxy-4-vinylphenol ^3^	1324	1.49 a	1.08 b	0.47 c	27.4 d	***
Butylated Hydroxytoluene ^3^	1513	0.49 a	0.7 a	0.25 b	n/d	***
**Carbonyl compounds**						
Decanal	1182	17.5 a	14.8 a	0 b	0 b	***
2-Dodecanol ^3^	1417	4.75 a	4.55 a	1.26 b	1.35 b	*
**Acetals**						
Diethyl acetal	730	7178 a	6561 a	2874 b	2750 b	*
1-Ethoxy-1-propoxyethane ^3^	755	24.9 a	18.3 a	13 a	0 b	*
1-Butoxy-1-ethoxyethane ^3^	875	21.7 a	19.8 a	14.9 a	39.4 b	*
1-(1-Ethoxyethoxy)pentane	977	133.2	123.3	112.7	140.4	ns
**Other compounds**						
Benzothiazole ^3^	1186	78.4	156.9	134.7	88.2	ns
Octane, 1,1′-oxybis- ^3^	1657	3.93	4.36	2.34	4.38	ns

^1^ Sig.: significance; *, **, and *** display the significance at 5%, 1%, and 0.5% by least significant difference; ns: not significant. Values with different superscript roman letters (a–d) in the same row are significantly different according to the Tukey’s range test (*p* < 0.05); ^2^ LRI—Linear Retention Index; ^3^ determined semi-quantitatively by measuring the relative peak area of each identified compound, according to the NIST [25] database, in relation to that of the internal standard.

**Table 5:** Terpenes in hopped wort and beer brewed with Marynka (M) or Lubelski (L) hop pellets, fermented by *L. thermotolerans* (Lt) or *S. cerevisiae* (Sc).

Compound [µg/L]	LRI ^2^	Wort		Lt		Sc		Sig. ^1^
		L	M	L	M	L	M	
Limonene	1027	0.08 ab	0.28 b	0.00 a	0.00 a	0.00 a	0.00 a	***
cis-Linalol oxide	1066	0.34 ab	0.28 ab	0.42 b	0.34 ab	0.41 b	0.18 a	*
Linalool	1092	1.21	0.80	0.98	0.80	1.15	0.44	ns
Perillen ^3^	1101	0.31 b	0.36 b	0.00 a	0.00 a	0.00 a	0.00 a	***
α-Terpineol	1171	0.84	0.69	0.70	0.68	0.46	0.65	ns
Citronellol	1210	0.00 a	0.00 a	0.88 ab	2.22 c	1.23 bc	3.50 d	***
Nerol ^3^	1218	0.59 b	1.06 c	0.00 a	0.00 a	0.00 a	0.00 a	***
Geraniol	1257	3.41 c	12.25 e	1.28 b	4.34 d	0.60 ab	0.34 a	***
Perilla alcohol ^3^	1295	0.74 c	0.41 b	0.20 a	0.09 a	0.00 a	0.00 a	***
β-Damascenone	1384	0.00 a	0.00 a	0.18 b	0.16 b	0.48 b	0.40 b	*
β-Caryophyllene	1414	1.06 bc	1.33 c	0.65 ab	0.62 ab	0.66 ab	0.27 a	***
Geranyl acetone ^3^	1443	0.83 ab	1.09 b	0.73 ab	0.54 a	0.65 ab	0.51 a	*
Humulene	1455	3.46 b	3.61 b	0.00 a	0.00 a	0.00 a	0.00 a	***
β-Farnesene ^3^	1462	2.43 b	3.12 b	0.70 a	0.76 a	0.57 a	0.54 a	***
α-Calacorene ^3^	1540	0.06 a	0.08 ab	0.13 bc	0.14 c	0.18 c	0.13 bc	***
Caryophyllene oxide	1573	1.40 b	1.85 c	0.19 a	0.20 a	0.00 a	0.00 a	***
Nerolidol ^3^	1562	0.00 a	0.00 a	0.71 b	0.63 b	0.42 b	0.41 b	***
Humulol ^3^	1600	0.70 b	0.71 b	0.44 a	0.31 a	0.47 a	0.31 a	*
Humulene epoxide II ^3^	1606	5.66 bc	6.50 c	3.66 ab	2.61 a	2.72 a	1.80 a	***
Patchulane ^3^	1618	2.78 b	2.91 b	1.65 a	1.56 a	1.71 a	1.23 a	***
Aromadendrene epoxide ^3^	1623	1.21 d	1.05 cd	0.71 bc	0.53 b	0.19 a	0.13 a	***
α-Muurolol ^3^	1632	0.51 b	0.60 b	0.24 a	0.20 a	0.17 a	0.12 a	***
τ-Muurolol ^3^	1646	1.69 d	2.14 d	0.89 c	0.85 bc	0.34 ab	0.28 a	***

^1^ Sig.: significance; * and *** display the significance at 5% and 0.5% by least significant difference; ns: not significant. Values with different superscript roman letters (a-d) in the same raw are significantly different according to the Tukey’s range test (*p* < 0.05); ^2^ LRI—Linear Retention Index; ^3^ determined semi-quantitatively by measuring the relative peak area of each identified compound, according to the NIST [25] database, in relation to that of the internal standard.

**Table 6:** The content of selected ions in wort brewed with Marynka (M) or Lubelski (L) hop pellets, and beer fermented by *L. thermotolerans* (Lt) or *S. cerevisiae* (Sc).

	L mg/L	M mg/L	Lt_L mg/L	Lt_M mg/L	Sc_L mg/L	Sc_M mg/L	Sig. ^1^
**Ca 422.7**	53.0	67.3	43.1 ± 5.04	62.8 ± 26.3	50.9 ± 3.4	42.6 ± 2.25	ns
**Fe 248.3**	10.0	12.1	9.9 ± 0.23	10.5 ± 0.1	10.3 ± 0.69	10.1 ± 0.13	ns
**Zn 213.9**	1.65	1.69	1.64 ± 0.19	1.72 ± 0.9	1.49 ± 0.25	1.09 ± 0.13	ns
**Mg 202.6**	197.9	199.7	147.21 ± 17.46	150.75 ± 21.9	168.82 ± 13.3	165.59 ± 3.01	ns

^1^ Sig.: significance; ns: not significant. Values with different superscript roman letters (a–b) in the same row are significantly different according to the Tukey’s range test (*p* < 0.05).
